# Partisan Differences in Legislators’ Discussion of Vaccination on Twitter During the COVID-19 Era: Natural Language Processing Analysis

**DOI:** 10.2196/32372

**Published:** 2022-02-18

**Authors:** Eden Engel-Rebitzer, Daniel C Stokes, Zachary F Meisel, Jonathan Purtle, Rebecca Doyle, Alison M Buttenheim

**Affiliations:** 1 Perelman School of Medicine University of Pennsylvania Philadelphia, PA United States; 2 Center for Emergency Care Policy and Research Philadelphia, PA United States; 3 Leonard Davis Institute of Health Economics Philadelphia, PA United States; 4 Department of Emergency Medicine Perelman School of Medicine University of Pennsylvania Philadelphia, PA United States; 5 Penn Injury Science Center University of Pennsylvania Philadelphia, PA United States; 6 Department of Public Health Policy and Management School of Global Public Health New York University New York City, NY United States; 7 Global Center for Implementation Science New York University New York City, NY United States; 8 School of Nursing University of Pennsylvania Philadelphia, PA United States

**Keywords:** social media, Twitter, vaccination, partisanship, COVID-19, vaccine, natural language processing, NLP, hesitancy, politicization, communication, linguistic, pattern

## Abstract

**Background:**

The COVID-19 era has been characterized by the politicization of health-related topics. This is especially concerning given evidence that politicized discussion of vaccination may contribute to vaccine hesitancy. No research, however, has examined the content and politicization of legislator communication with the public about vaccination during the COVID-19 era.

**Objective:**

The aim of this study was to examine vaccine-related tweets produced by state and federal legislators during the COVID-19 era to (1) describe the content of vaccine-related tweets; (2) examine the differences in vaccine-related tweet content between Democrats and Republicans; and (3) quantify (and describe trends over time in) partisan differences in vaccine-related communication.

**Methods:**

We abstracted all vaccine-related tweets produced by state and federal legislators between February 01, 2020, and December 11, 2020. We used latent Dirichlet allocation to define the tweet topics and used descriptive statistics to describe differences by party in the use of topics and changes in political polarization over time.

**Results:**

We included 14,519 tweets generated by 1463 state legislators and 521 federal legislators. Republicans were more likely to use words (eg, “record time,” “launched,” and “innovation”) and topics (eg, Operation Warp Speed success) that were focused on the successful development of a SARS-CoV-2 vaccine. Democrats used a broader range of words (eg, “anti-vaxxers,” “flu,” and “free”) and topics (eg, vaccine prioritization, influenza, and antivaxxers) that were more aligned with public health messaging related to the vaccine. Polarization increased over most of the study period.

**Conclusions:**

Republican and Democratic legislators used different language in their Twitter conversations about vaccination during the COVID-19 era, leading to increased political polarization of vaccine-related tweets. These communication patterns have the potential to contribute to vaccine hesitancy.

## Introduction

As of December 2021, the COVID-19 pandemic has resulted in over 45 million infections and 780,000 deaths in the United States [1]. Despite the high death toll attributed to the pandemic and the emergence of safe and effective vaccines, vaccine hesitancy, particularly in Republican-leaning states, remains a significant obstacle to achieving the estimated 70% population immunity required to reach herd immunity [1-3].

It has been hypothesized that this geographic variation in vaccination may be the result of the politicization of public health topics during the COVID-19 pandemic [4]. Survey evidence from early in the pandemic suggests that such politicization may have resulted in members of the public interpreting COVID-19–related risk and adopting preventive health measures in partisan ways [5]. Consistent with these findings, geolocation data have revealed lower rates of social distancing in counties that supported Donald Trump in the 2016 election compared with counties that did not [6]. There is also evidence that these partisan differences extend to opinions about the SARS-CoV-2 vaccine. Prior research has established lower rates of vaccination among Republicans compared with those of Democrats and has found that this partisan gap in vaccination increased throughout the COVID-19 pandemic [7,8]. This gap is not explained by demographic differences, differences in institutional trust, or differences in the level of concern about the pandemic, suggesting that partisan identity, in and of itself, may be informing individuals’ health care decisions and driving differences in vaccine sentiment [7].

The politicization that has characterized the COVID-19 era is especially concerning because there is evidence that politicized vaccine-related communication may contribute to vaccine hesitancy [9-12]. For example, a study of human papillomavirus (HPV) vaccination found that exposure to real-world politicized discussion about the vaccine was associated with decreased support for immunization programs and reduced trust in doctors and government [9]. A similar experimental study found that respondents exposed to a news brief that included political conflict about the HPV vaccine were less likely to support a vaccine mandate compared with those exposed to a news brief without controversy [10]. Other studies have found similar associations between politicized discussion of vaccination and decreased support of vaccine mandates and intention to vaccinate [11,12]. These findings suggest that the language politicians use to communicate with their constituents about vaccination during the COVID-19 pandemic may play an important role in determining vaccine uptake.

The existing research has established partisan differences in the way that political figures communicated with the public about SARS-CoV-2 [13-15]. Much less research projects, however, have examined communication from political leaders about vaccination (and partisan differences in that communication) during the COVID-19 pandemic. This is an important gap in the literature for several reasons. First, experimental evidence suggests that politicians’ Twitter activity and communication with the public not only reflect the opinions of constituents but also have the ability to shape the vaccine perspectives of their followers [16,17]. For example, a study using tweets from former President Trump found that exposure to antivaccine tweets generated by Trump led to an increase in vaccine concern among his followers [17]. Communication about vaccination from state and federal legislators is also of particular importance given that, in addition to communicating with their constituents, these legislators enact policies that impact vaccine development and distribution. Despite the importance of legislators’ communication about vaccination to the public, existing research on vaccine-related Twitter activity has primarily focused on partisan trends among the general public. No prior research has characterized the vaccine-related Twitter activity of state and federal legislators or partisan differences in such communications during the COVID-19 vaccine development process.

We previously found that the arrival of the COVID-19 pandemic was associated with a dramatic increase in the volume of legislator’s vaccine-related tweets [18]. Here, we build on that previous work by characterizing the content, not just the volume, of Twitter discourse about vaccination from legislators during the COVID-19 vaccine development process. The objective of this study was to examine vaccine-related tweets produced by state and federal legislators during the COVID-19 era to (1) describe the content of vaccine-related tweets; (2) examine differences in vaccine-related tweet content between Democrats and Republicans; and (3) quantify (and describe trends over time in) partisan differences in vaccine-related communication.

## Methods

### Data

We used Quorum, a public affairs software platform that stores policy-related documents, to gather all vaccine-related tweets produced by state or federal legislators between February 1, 2020, and December 11, 2020. We defined February 1, 2020, as the arrival date of COVID-19 in the United States based on the United States’ declaration of a public health emergency (January 31, 2020) and restriction of global air travel (February 2, 2020) [19]. We selected December 11, 2020, as the endpoint of our data collection because it was the date of the first Food and Drug Administration emergency use authorization for a COVID-19 vaccine [19]. While some legislators maintain both personal and professional Twitter accounts, only the tweets generated from professional Twitter accounts were used in this study.

We defined tweets as vaccine-related if they contained any of the following terms in the body of the tweet or retweet: “vaccine,” “vaccination,” “immunization,” “vax(x),” “antivax(x),” “anti-vax(x),” “antivax(x)er,” “anti-vax(x)er,” “vax(x)ine,” “in(n)oculate,” “in(n)oculation.” This term list was generated based on a review of search terms in the existing literature about vaccine sentiment on Twitter [20-22]. One author manually reviewed all tweets generated by this search, and any tweets that were unrelated to human vaccination were removed. This study was exempt from Institutional Review Board approval due to the public availability of the data.

### Measures

Legislators’ political party was abstracted from Quorum. Tweets were defined as related to COVID-19 if they contained a word or phrase related to the disease (eg, “coronavirus” or “SARS-CoV-2”). Tweets were defined as discussing a non–COVID-19 disease if they mentioned any infectious disease other than COVID-19 (eg, “MMR” or “influenza”). A complete list of infectious disease-related terms used in the data set was compiled during a manual review of the data and was used to build these variables (Multimedia Appendix 1). We used tweet topics to quantify the political polarization of vaccine-related communication by calculating the sum of the absolute difference in topic prevalence for all tweet topics per month, as previously described [23].

### Descriptive and Bivariate Analysis

We used summary statistics to describe tweet frequency and characteristics of included tweets (ie, mentions of COVID-19 versus non–COVID-19 infectious diseases, the percent of tweets generated by each political party, and the frequency of tweets versus retweets). In order to further characterize differences in vaccine-related Twitter activity between Republicans and Democrats, we used chi-square tests to describe the relationship between political party and tweet characteristics. The tweet characteristics examined in this study were (1) mentions of COVID-19 versus non–COVID-19 infectious disease and (2) whether each tweet was an original tweet or a retweet. Descriptive analyses were conducted using Stata statistical software, version 16.1 (Stata Corp).

### Natural Language Processing Analysis

**Figure 1:**
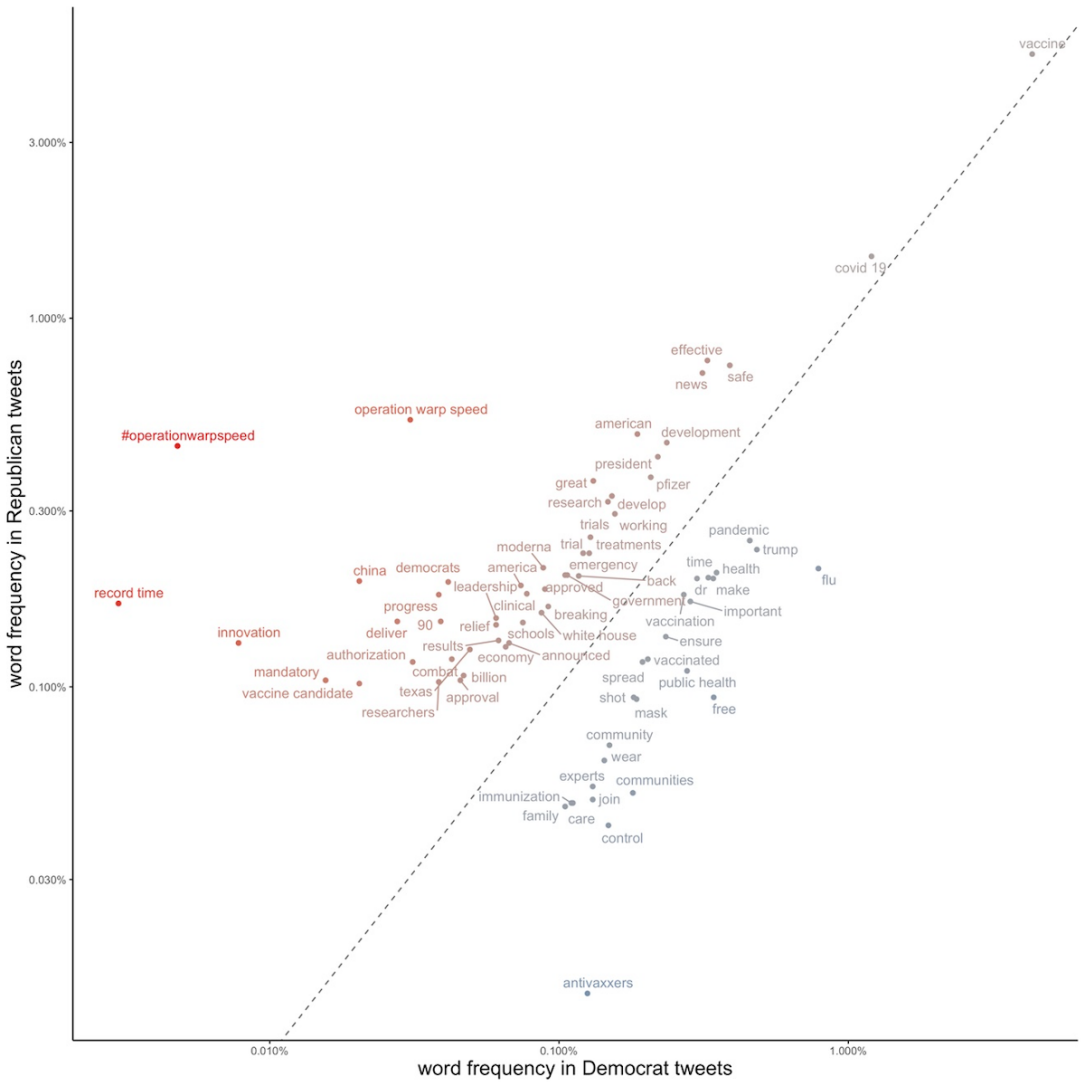
Word and term frequency in vaccine tweets for Democrats vs Republicans.

We identified all words and 2-word phrases appearing with a frequency of at least 0.1% across tweets by Democrats or Republicans. We used chi-square testing (*P* value cut-off of Bonferroni corrected *P*<.001) to identify words used with significantly different frequency between the 2 parties. We plotted words by frequency of use in Democrat versus Republican tweets (Figure 1). To account for language changes that occurred within the COVID-19 era, we repeated this process for all 3 waves of the COVID-19 pandemic (Multimedia Appendix 2). We defined the start and end dates of each wave based on the nadir of the 7-day moving average of new cases in the United States [24].

In order to describe trends in tweet content over time, we used latent Dirichlet allocation (LDA) to define the topic or topics of each tweet [25]. LDA is a topic modeling approach that defines topics based on cooccurring words across tweets, excluding common words. The number of topics defined by LDA (in this case, 25) was selected iteratively through a combination of algorithmic coherence scores and manual review of topic interpretability, conducted by 2 authors. Each tweet could then be described by a unique probability distribution of the 25 topics.

Three authors evaluated each topic by manually reviewing the 10 words and 10 tweets most closely associated with that topic. The topics that all 3 authors agreed had a coherent meaning were included in the final analysis (20 topics total). To confirm topic interpretability, 3 authors manually checked each of these 20 topics against an additional 20 randomly selected tweets associated with each topic [23]. We used summary statistics to describe mean topic representation, defined as the mean topic probability across all tweets from a given party and time period and multiplied by 100%. We used Wilcox signed-rank tests (*P* value cut-off of Bonferroni corrected *P*<.001) to compare mean topic representation by political party. We conducted natural language processing analyses and generated figures using R version 4.0.3 (R Foundation for Statistical Computing).

## Results

We included a total of 14,519 vaccine-related tweets. Of these tweets, 61.8% (n=8968) were generated by Democrats, 37.2% (n=5401) were generated by Republicans, and 1.0% (n=150) were generated by third-party or nondesignated legislators. The sample included 5653 (38.9%) retweets. The majority of tweets (55.1% [n=7996]) contained a COVID-19–related term, and 11.8% of tweets (n=1706) referenced a non–COVID-19 infectious disease (eg, measles and influenza). The included tweets were generated by 1984 unique legislators. The majority of the included legislators were state representatives (73.7% [n=1463]) as opposed to federal representatives (26.3% [n=521]). In terms of political party, 63.7% (n=1264) of the included legislators were Democrats, 35.1% (n=696) were Republicans, and 1.2% (n=24) were independent or undesignated.

Vaccine-related tweets generated by Republicans were less likely than vaccine-related tweets generated by Democrats to be retweets (36.7% [n=1992] for Republicans versus 40.3% [n=3614] for Democrats; *P*<.001). Republican vaccine tweets were also less likely than Democratic vaccine tweets to reference a non–COVID-19 disease (7.5% [n=404] for Republicans versus 14.4% [n=1289] for Democrats; *P*<.001), and more likely to reference COVID-19 (58.3% [n=3146] for Republicans versus 53.2% [n=4770] for Democrats; *P*<.001).

**Figure 2:**
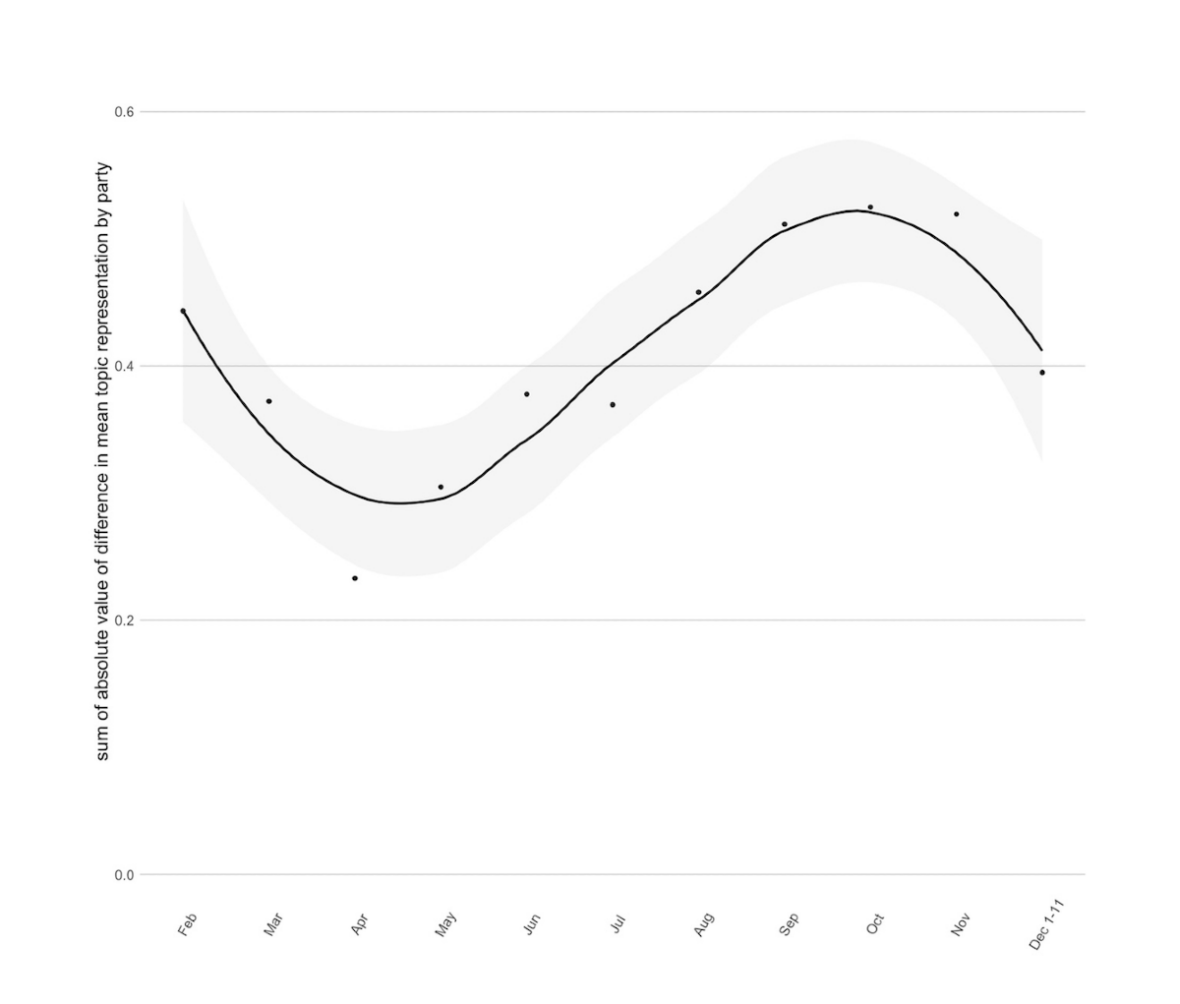
Trends in partisanship over time (defined as the sum of absolute difference in mean topic representation across parties by month).

Words and phrases more commonly used among Republicans (vs Democrats) in vaccine-related tweets included “operation warp speed,” “record time,” “innovation”, and “China.” Words and phrases more frequently used among Democrats (vs Republicans) included “anti-vaxxers,” “flu,” “communities,” “public health,” and “free” (Figure 2). To account for language changes over the study period, we repeated this analysis separately during each phase of the pandemic (Multimedia Appendix 2). During the first wave of the pandemic, words that were strongly associated with Republicans included “clean-funding,” “cares act,” and “innovation.” During the second and third wave, keywords associated with Republicans included words related to Operation Warp Speed (eg, “record time,” “launched,” “ingenuity,” “#OperationWarpSpeed,” and “innovation”) as well as the word “mandate.” During the first and second wave of the pandemic, terms strongly associated with Democrats included language supporting vaccines and opposing the antivaccine movement (eg, “#VaccinesWork,” “#DoctorsSpeakUp,” and “#IVaxToProtect”). In wave 3 of the pandemic, the term most strongly associated with Democrats was “Meadows” (referring to former White House chief of staff Mark Meadows). Across all 3 waves, there were more terms strongly associated with Republicans than Democrats (Figure 1 and Multimedia Appendix 2).

We included 20 topics in our final analysis (Tables 1-3). The topics with the highest percent topic representation were (1) Operation Warp Speed success; (2) vaccine effectiveness; (3) COVID-19 vaccine updates; (4) COVID-19 relief package content; and (5) nonpharmaceutical interventions as a bridge to vaccine. The topics that were more prevalent among Republicans included (1) Operation Warp Speed success; (2) COVID-19 vaccine updates; (3) international efforts to hack vaccine-related research; and (4) vaccine effectiveness. The topics that were more prevalent among Democrats included (1) vaccine prioritization; (2) children and parents; (3) reliance on vaccine as pandemic solution; (4) local, free, non–COVID-19 vaccine clinics; (5) nonpharmaceutical interventions as a bridge to vaccine; (6) influenza; (7) state and local vaccine distribution plans; and (8) discussion of antivaxxers. The remaining topics were equally prevalent between Democrats and Republicans (Tables 1-3; significance was defined as Bonferroni corrected *P*<.001, and topics in each section are listed in order of decreasing magnitude of partisan difference).

**Table 1:** Topics with significantly higher mean representation among Republicans.

Topic name	Keywords	Representative tweets (links and retweet handles removed for clarity)	Percent Democratic topic representation	Percent Republican topic representation
Operation Warp Speed success	operationwarpspeed, safe, effective, american, covid, president, develop, working, deliver, progress	“Under @realDonaldTrump leadership, Operation Warp Speed will deliver a safe and effective vaccine in record time!”	2.4	12.3
COVID-19 vaccine effectiveness	news, covid, pfizer, effective, great, breaking, moderna, coronavirus, pfizers, emergency	“Massively good news here. The Associated Press (@AP): BREAKING: Pfizer says early data signals its vaccine is effective against COVID-19; on track to seek U.S. review later this month.”	3.4	6.8
COVID-19 vaccine updates	covid, trials, trial, clinical, phase, coronavirus, results, news, good, data	“Promising news from Oxford on a vaccine!”	3.8	6.4
International efforts to hack vaccine-related research	coronavirus, research, covid, china, global, world, develop, working, find, work	“U.S. to Warn That China Is Attempting to Steal Coronavirus Vaccine Research”	3.1	4.5

**Table 2:** Topics with significantly higher mean representation among Democrats.

Topic name	Keywords	Representative tweets (links and retweet handles removed for clarity)	Percent Democratic topic representation	Percent Republican topic representation
Influenza	flu, protect, shot, important, vaccinated, year, people, learn, fluseason, covid	“The best way to protect against the flu this season is to get vaccinated.”	5.9	2.6
Discussion of “anti-vaxxers”	antivaxxers, publichealth, science, protect, antivaccine, antivax, ivax, misinformation, stopantivaxviolence, vaccineswork	“Anti-vaxxers. Anti-maskers. Pro-disease.”	4.6	2.0
Local, free, non–COVID-19 vaccine clinics	free, flu, health, immunization, school, vaccinations, county, clinic, call, today	“Need your flu shot and other vaccinations? Register by October 15 for a drive-thru flu shot clinic I am co-hosting with @RepDanMiller”	4.7	2.1
NPI^a^ as a bridge to vaccine	mask, covid, masks, wear, continue, cases, spread, stay, wearing, hands	“We must continue to practice caution and #MaskUpPA. There is still no vaccine, so we must be careful.”	5.5	3.3
State and local vaccine distribution plan	covid, distribution, plan, communities, ensure, states, texas, distribute, black, state, vaccination	“I wrote a letter to the Task Force on Infectious Disease Preparedness and Response to ensure minorities & communities disproportionately impacted by #COVID19 are not left behind in a #vaccine allocation & distribution plan. #txlege #ElPaso”	5.2	3.4
Reliance on vaccine as pandemic solution	pandemic, control, biden, virus, trump, testing, joe, lives, coronavirus, president	Mark Meadows: “We’re not going to control the pandemic, we are going to control the fact that we get vaccines, therapeutics and other mitigations.” Jake Tapper: “Why aren’t we going to get control of the pandemic?” Meadows: “Because it is a contagious virus” #CNNSOTU	4.1	2.9
Children and parents	children, kids, vaccination, parents, vaccinate, diseases, time, vaccinations, medical, protect	“Parents! Make sure that your child's immunizations are up-to-date as part of your back-to-school preparations. Vaccines are a necessary precaution needed to protect infants, children and teens from serious childhood diseases. Learn more”	4.2	3.1
Vaccine prioritization	state, people, covid, line, teachers, healthcareworkers, session, business, essential, pandemic	“A vaccine needs to go to our health care workers, first responders, and those most vulnerable 1st, the Legislature can wait.”	3.5	2.5

^a^NPI: nonpharmaceutical interventions.

**Table 3:** Topics with no significant difference in mean representation by party.

Topic name	Keywords	Representative tweets (links and retweet handles removed for clarity)	Percent Democratic topic representation	Percent Republican topic representation
COVID relief package debate	testing, relief, schools, families, covid, democrats, senate, americans, smallbusinesses, funding	“Resources to get kids back to school or child care Dems blocked it Resources to protect workers' paychecks Dems blocked it Resources for vaccines & testing Dems blocked it Resources for another round of job-saving Paycheck Protection Program loans Dems blocked it”	2.8	4.8
Impact of political pressure on vaccine safety	fda, covid, safety, science, emergency, dr, confidence, process, americans, political	“Even if political pressure didn't rush a COVID19 vaccine, the mere perception among a majority of Americans that it did undermines public trust. We must prevent the vaccine from being unsafely rushed & Americans from having reason to distrust its safety.”	4.6	3.9
Production, distribution, and rollout	covid, doses, receive, end, pfizer, million, ready, week, residents, coronavirus	“COVID-19 vaccine could be in Missouri as early as Dec. 15, 2020.”	3.7	4.3
COVID-19 updates, press conferences, and town halls	covid, dr, today, join, discuss, watch, pm, latest, update, questions	“US_FDA Commissioner, @SteveFDA, M.D., will join us for today's Instagram live. We will discuss the progress of a #COVID19 vaccine. Be sure to watch at 2 p.m. on my Instagram page, @SenatorTimScott.#LiveWithTim”	4.6	4.1
President Trump	trump, president, people, fauci, dr, donald, realdonaldtrump, election, time, coronavirus	“He has completely divorced himself from reality and that's why the death of almost 195,000 Americans doesn't phase him. TRUMP: It is going away STEPHANOPOULOS: Without a vaccine? TRUMP: Sure. Over a period of time S: And many deaths TRUMP: It's gonna be herd developed”	4.2	3.7
COVID-19 relief package content	coronavirus, development, billion, funding, treatments, passed, local, bill, response, research	“Yesterday, I voted to support an emergency funding package to tackle #Coronavirus at home & abroad, including resources for state & local health departments and expedited vaccine development.”	5.3	4.8
Vaccine profiteering	covid, make, government, people, coronavirus, dr, trump, stock, americans, working	“Trump's New COVID-19 Czar Holds $10 Million In Vaccine Company Stock Options”	3.5	3.2
Things government can (and cannot) do to increase vaccine uptake	covid, free, bill, healthcare, act, care, health, access, support, treatment	“It is one thing to have a vaccine - it is another to be able to effectively distribute it to people across the country. We must put #FamiliesFirst and pass a bipartisan relief bill that ensures additional funding for vaccine distribution.”	3.4	3.2

Polarized partisan communication decreased between February and April 2020 but increased for most of the study period (May through November) before trending down slightly during the first 11 days of December 2020. The increase in polarized communication was driven by several topics that demonstrated a widening gap in mean topic representation by political party over the study period. The topics that demonstrated a widening partisan gap with higher representation among Democrats included (1) President Trump; (2) influenza; (3) local, free non–COVID-19 vaccine clinics; and (4) state and local vaccine distribution plans. The topics that demonstrated a widening partisan gap with higher representation among Republicans included (1) Operation Warp Speed success and (2) COVID-19 relief package debate (Multimedia Appendix 3).

**Figure 3:**
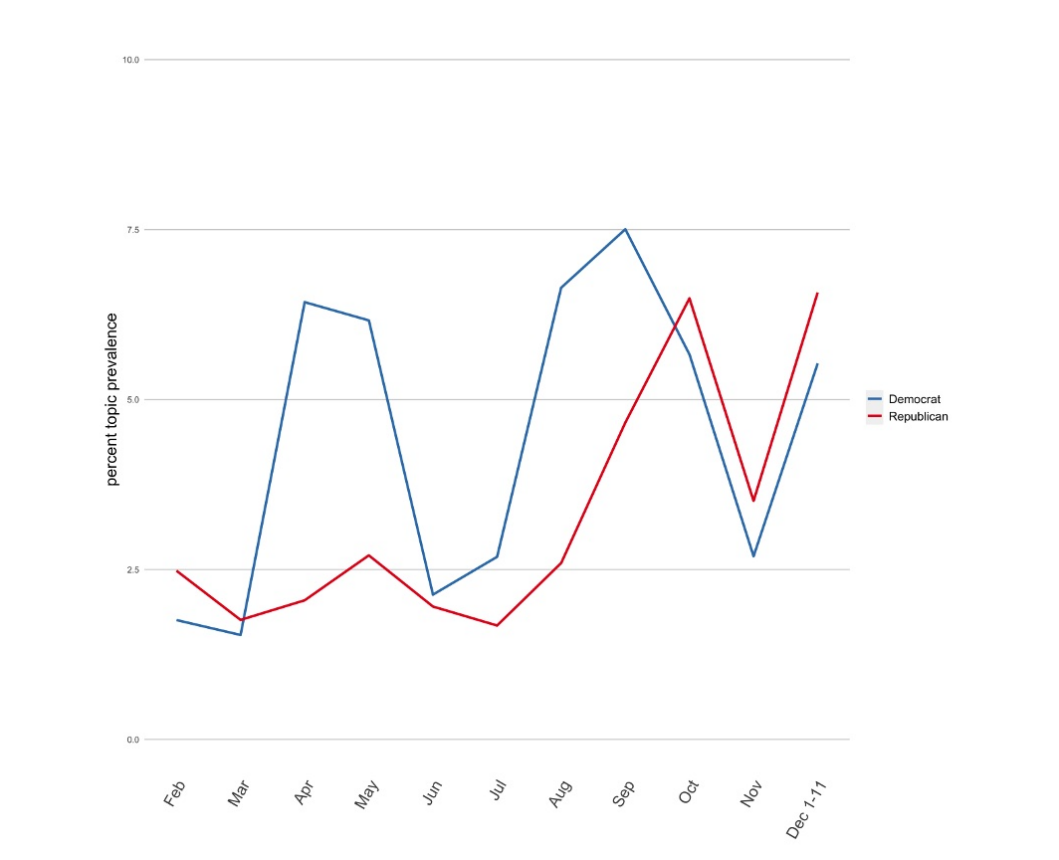
Partisan trends in Topic 5 (impact of political pressure on vaccine safety) over time.

Several topics demonstrated decreasing partisan gaps over the study period, including the impact of political pressure on vaccine safety. While Democrats were more likely to discuss this topic early on, Republican engagement with this topic increased to match that of Democrats toward the end of the study period (Figure 3). Topics that remained relatively nonpartisan over time (ie, had similar mean topic representation at each time point) included (1) vaccine prioritization; (2) production, distribution, and rollout; and (3) COVID-19 relief package content (Multimedia Appendix 3).

## Discussion

### Principal Findings

We examined vaccine-related Twitter communication from state and federal legislators during the COVID-19 pandemic. We found that Republicans and Democrats used different words, phrases, and topics to discuss vaccination during the COVID-19 era. Republicans discussed vaccination using a narrow set of topics focused on progress toward the development of the SARS-CoV-2 vaccine. Democrats, on the other hand, were engaged in a more wide-ranging conversation covering a broad set of vaccine-related topics that were aligned with public health messaging related to the vaccine. We also identified patterns in legislator discussion of vaccination (eg, increased partisanship and discussion of the impact of political pressure on vaccine safety) that have the potential to contribute to SARS-CoV-2 vaccine hesitancy.

The language used by Republican legislators about vaccination during the COVID-19 era was narrowly focused on the successful development of a SARS-CoV-2 vaccine. This was illustrated in both the keywords (eg, “record time,” “launched,” and “innovation”) and topics (eg, Operation Warp Speed success and vaccine effectiveness) that were associated with Republicans. Overall, fewer topics were associated with Republicans, and the keywords used by Republicans were more highly partisan than those used by Democrats. Both findings are consistent with the use of more focused, consistent messaging in the Republican party. In addition, Republicans were more likely than Democrats to explicitly reference COVID-19 in their tweets and were almost half as likely as Democrats to discuss vaccination for non–COVID-19 infectious diseases. This is consistent with our previous paper in which we demonstrated that, prior to COVID-19, Republican legislators were only minimally engaged in Twitter discussion about vaccination, but their engagement increased markedly with the arrival of the pandemic [18]. We hypothesized that Republican vaccine engagement may have increased because the development of a SARS-CoV-2 vaccine during a Republican presidency would represent a political victory for the party [24]. The narrow focus on Operation Warp Speed (as opposed to vaccine hesitancy, flu vaccination, or other important vaccine-related topics) described in this paper is consistent with that hypothesis. The political stakes of successful vaccine development may have been further increased by a Republican desire for an “October surprise” given that the topic Operation Warp Speed success rose in mean representation in the months leading up to the presidential election [26]. This raises the concerning implication that, with the resolution of COVID-19, Republicans may return to relative disengagement with the topic of vaccination.

Democrats used a broader set of topics to discuss vaccination during the COVID-19 era. Democrats were more likely to tweet about non–COVID-19 infectious diseases and tweeted about a larger number of topics than Republicans. They used a wide range of keywords (eg, “anti-vaxxers,” “flu,” “communities,” and “free”) and topics (eg, distribution of a successful vaccine, the antivaccine movement, vaccination for non–COVID-19 infectious diseases, the importance of utilizing other public health measures until a successful vaccine, and more) to discuss vaccination. These topics were also more consistent with COVID-19–related public health messaging in the lay and academic press, much of which discussed vaccine affordability, the ongoing importance of non–COVID-19 vaccines, vaccine distribution and access, and concerns about vaccine hesitancy [27-30]. The similarity between Democratic legislators’ messaging and public health messaging about the COVID-19 vaccine is consistent with the existing research. A recent study using vaccine-related Twitter data from the general public demonstrated an increase in social connection and signal boosting between Democrats and public health organizations following the arrival of the pandemic [31]. These results are also consistent with a broader literature that suggests that Democrats may be more likely than Republicans to defer to scientific authority [32,33]. Our findings may also help to explain partisan differences in intention to vaccinate. Democratic legislators’ vaccine-related tweets were more consistent with public health messaging than those of Republicans. As a result, followers of Democratic politicians may have been exposed to higher quality information related to COVID-19 vaccination, which may contribute to the partisan gap in willingness to accept the COVID-19 vaccine.

In this study, we also described patterns of vaccine-related communication from legislators that have the potential to contribute to vaccine hesitancy. The COVID-19 pandemic created an opportunity for either (1) mobilization of political leaders around a shared understanding of the importance of the vaccine or (2) an increase in polarization of the already politically polarized topic of vaccination. While there was a nadir in polarization of vaccine-related communication early in the pandemic (April 2020), the bulk of the study period was notable for increased polarization among federal and state legislators. This finding is concerning given literature suggesting that polarization in vaccination discussion may contribute to vaccine hesitancy [9,10]. Previous research by Fowler and Gollust [9] on the politicization of the HPV vaccine found that once a public health issue was politicized, it tended to remain so and failed to return to its previous baseline of politicization. In the case of this study, this finding implies that even if polarization decreases in the coming months, vaccines may remain more politicized than they were before the pandemic. Concern has also been raised in the literature that hesitancy about a specific vaccine may lead to decreased uptake of unrelated vaccines [34]. This phenomenon could further compound any harm inflicted by the politicization of the COVID-19 vaccine.

In addition to the rise in politically polarized communication during the study period, we also noted the emergence of topics that have been associated with mistrust of vaccines. For example, the topic “Impact of political pressure on vaccine safety” was initially primarily discussed by Democrats. However, by the second half of the pandemic, Republicans had joined the conversation, and the topic was again increasing in mean representation. This finding is concerning given experimental evidence that suggests that exposure to this topic may be associated with decreased belief in the importance of the COVID-19 vaccine [35]. Survey data have also demonstrated that most Americans are very or somewhat worried that the Food and Drug Administration would rush a COVID-19 vaccine in response to political pressure. Similarly, the topic of “Vaccine profiteering” has been found to be associated with increased mistrust of the COVID-19 vaccines [36]. The emergence of these themes in legislators’ Twitter activity has the potential to further legitimize and contribute to this public concern and mistrust, resulting in vaccine hesitancy.

The use of natural language processing methods for monitoring politicians’ communication may have implications for improving the quality of public health-related messages on Twitter. This is especially relevant given the increasing pressure on social media platforms to monitor public officials’ discourse following President Trump’s use of misinformation during the COVID-19 pandemic and eventual deplatforming [37]. The close monitoring of how politicians discuss public health issues is especially important in light of recent findings that politicians are more likely than scientists to appear in COVID-19–related newspaper coverage [38].

While Twitter has been used to study legislator communication about COVID-19, to our knowledge, this is the first study to examine how legislators used Twitter to communicate with the public about vaccination in the COVID-19 era [39]. Other strengths of this study include the longitudinal nature of our data and the uniquely important subpopulation of Twitter users examined in this analysis. We also note some limitations to this study. While Twitter is an important way that legislators engage with the public, many choose to engage with constituents using other platforms. As a result, this study does not capture the full scope of legislator communication with the public. There are also limitations to the natural language processing methods. While we were able to capture differences by party in the use of topics, we were unable to capture partisan differences in tone during the discussion of a given topic. For example, tweets endorsing or criticizing former President Trump’s pandemic response would both fall into the “President Trump” topic. As a result, our polarization metric may underestimate the actual differences in vaccine discussion by party.

### Conclusion

Republican and Democratic legislators engaged in substantively different conversations about vaccination on Twitter during the COVID-19 era, which led to an increase in political polarization of vaccine-related tweets throughout much of the pandemic. Republicans were engaged in a focused conversation about the successful development of a vaccine, and Democrats used a broader range of topics, which was more consistent with public health messaging about vaccination. These patterns have the potential to contribute to vaccine hesitancy, and future research is needed to determine the real-world impact of political communication on COVID-19 vaccine uptake.

## Appendix

Multimedia Appendix 1 List of COVID-19 disease terms and non–COVID-19 disease terms.

Multimedia Appendix 2 Word and term frequency in vaccine tweets for Democrats vs Republicans by pandemic period (limited to the 30 most significantly different terms by party for ease of viewing).

Multimedia Appendix 3 Mean percent topic representation over time among Democratic and Republican posts for all included topics (5 topics did not reflect a coherent theme and were thus excluded).

## Abbreviations

HPV: human papillomavirus
LDA: latent Dirichlet allocation
