# Supplementary material for: Partisan Differences in Legislators’ Discussion of Vaccination on Twitter During the COVID-19 Era: Natural Language Processing Analysis
Source: JMIR Infodemiology. 2022 Feb 18;2(1):e32372. doi: 10.2196/32372 (PMC8862742; doi:10.2196/32372)
Supplement: Multimedia Appendix 1 [file infodemiology_v2i1e32372_app1.pdf]

S1 Table. List of COVID-19 disease terms and non-COVID-19 disease terms.

COVID-19 terms	Non-COVID-19 infectious disease terms
C19	AIDS
Corona	Chicken( )pox
Corona( )virus	Cholera
COVID	Dengue Fever
COVID(-)19	Diphtheria
CV	DTaP
CV19	Ebola
Rona	Flu
SARS(-)CoV(-)2	H1N1
Sarscov	HCV
	Hepatitis
	HIV
	HPV
	Influenza
	Malaria
	Measles
	Measles-Mumps-Rubella
	Meningitis
	Meningococcal
	MERS
	MMR
	Mumps
	Pertussis
	Pneumonia
	Polio
	Rabbit fever
	Rabies
	Rubella
	SARS
	Shingles
	Smallpox
	Swine flu
	TB
	Tdap
	Tetanus
	Tuberculosis
	Tularemia
	Typhoid
	Whooping cough
	Yellow fever
