# Supplementary material for: Partisan Differences in Legislators’ Discussion of Vaccination on Twitter During the COVID-19 Era: Natural Language Processing Analysis
Source: JMIR Infodemiology. 2022 Feb 18;2(1):e32372. doi: 10.2196/32372 (PMC8862742; doi:10.2196/32372)
Supplement: Multimedia Appendix 2 [file infodemiology_v2i1e32372_app2.pdf]

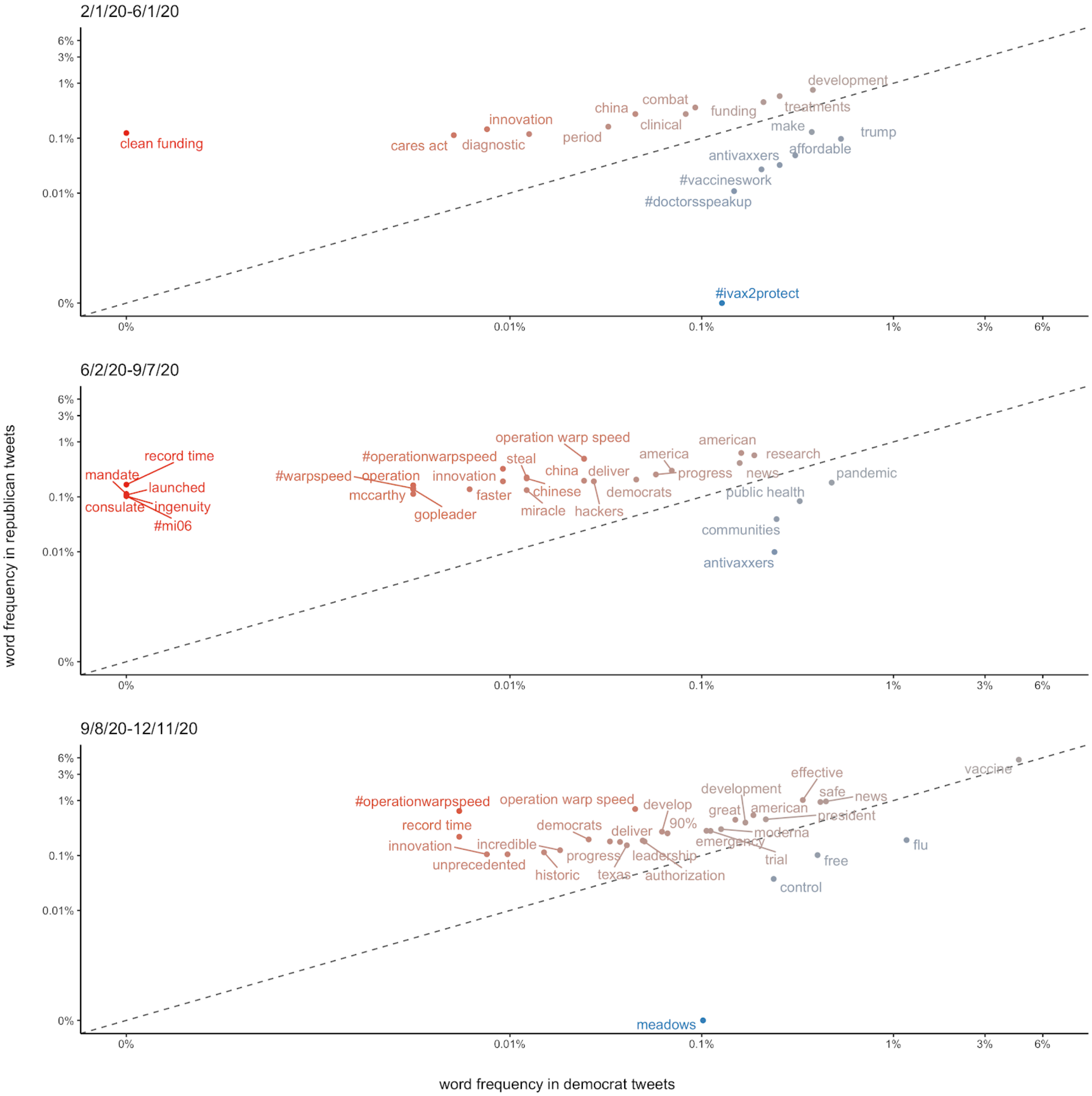

S1 Fig. Word and term frequency in vaccine tweets for Democrats vs. Republicans by pandemic period[a]

[a]Supplemental Figure 1 was limited to the 30 most significantly different terms by party per time period for ease of viewing.
