# Supplementary material for: Partisan Differences in Legislators’ Discussion of Vaccination on Twitter During the COVID-19 Era: Natural Language Processing Analysis
Source: JMIR Infodemiology. 2022 Feb 18;2(1):e32372. doi: 10.2196/32372 (PMC8862742; doi:10.2196/32372)
Supplement: Multimedia Appendix 3 [file infodemiology_v2i1e32372_app3.pdf]

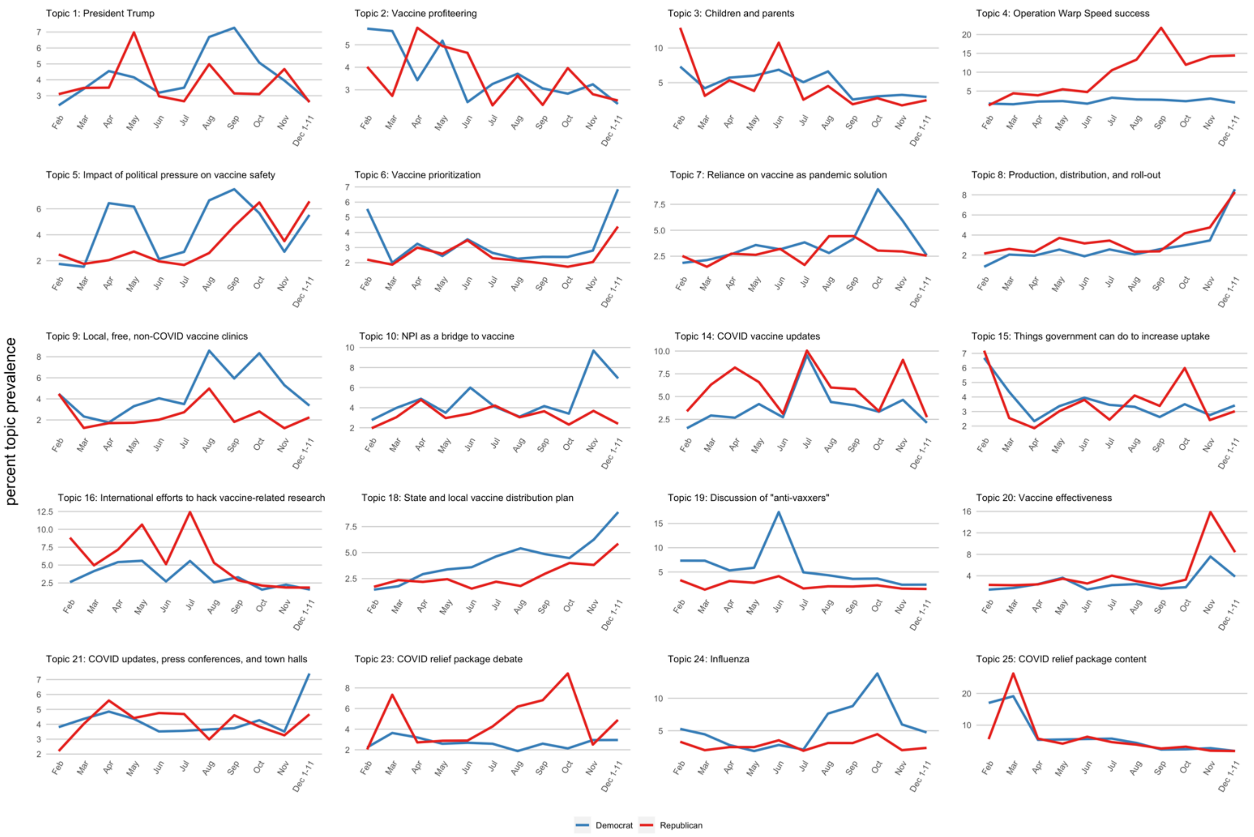

S2 Fig. Mean percent topic representation over time among Democratic and Republican posts for all included topics*

*Five topics were determined on manual review to not reflect a coherent theme and are excluded from this figure
